# Supplementary material for: Sub-nanosecond all-optically reconfigurable photonics in optical fibres
Source: Nat Commun. 2025 Jul 19;16:6665. doi: 10.1038/s41467-025-61984-8 (PMC12276229; doi:10.1038/s41467-025-61984-8)
Supplement: Supplementary file 1 — Supplementary Information [file 41467_2025_61984_MOESM1_ESM.pdf]

SUPPLEMENTARY INFORMATION
Sub-nanosecond all-optically reconfigurable photonics in optical fibres

KUNHAO JI[1,*], DAVID J. RICHARDSON[1,2], STEFAN WABNITZ[3], AND MASSIMILIANO GUASONI[1,*]

[1]*Optoelectronics Research Centre, University of Southampton, Southampton SO17 1BJ, United Kingdom*
[2]*Microsoft (Lumenisity Limited), Unit 7, The Quadrangle, Abbey Park Industrial Estate, Romsey, SO51 9DL, United Kingdom*
[3]*Department of Information Engineering, Electronics and Telecommunications (DIET), Sapienza University of Rome, 00184 Rome, Italy*
*[*m.guasoni@soton.ac.uk](mailto:m.guasoni@soton.ac.uk)*

Supplementary Information 1: Fibre modes and coefficients

In Fig. S1, we present the spatial profiles of the modes of the fibres tested in our experiments: a homemade dual-core fibre (DCF) and three-core fibre (TCF) supporting respectively 2 and 3 guided modes; and then a polarization-maintaining (PM) few-mode fibre (PM1550-xp from Thorlabs) and a highly nonlinear PM few-mode fibre (PMHN1 from Thorlabs) supporting 3 guided modes, as well as a PM few-mode fibre (PM2000 from Thorlabs) supporting 6 guided modes. Note that, in our experiments, the DCF is used both as bimodal fibre to illustrate multimode manipulation (Fig. 3 of the manuscript) and as multicore fibre for multicore manipulation (Fig. 5 of the manuscript). Modes M_1 and M_2 in the DCF correspond to the supermodes with the core fields in phase and anti-phase, respectively. Modes M_1, M_2, and M_3 in the PM1550-xp and PMHN1 fibre correspond to the standard linearly polarized modes LP_{01}, LP_{11e}, and LP_{11o}, respectively. Modes M_1, M_2, M_3, M_4, M_5, and M_6 in the PM2000 fibre correspond to the standard linearly polarized modes LP_{01}, LP_{11e}, LP_{11o}, LP_{21e}, LP_{21o}, and LP_{02}, respectively. Note that in these fibres $LP_{11e}(LP_{21e})$ and $LP_{11o}(LP_{21o})$ are non-degenerate.

Tables S1 and S2 list the Kerr coefficients and inverse group velocities for the fibres under test. These coefficients, along with the spatial profiles of the modes, were computed using finite element method simulations (central wavelength λ_c =1040 nm).

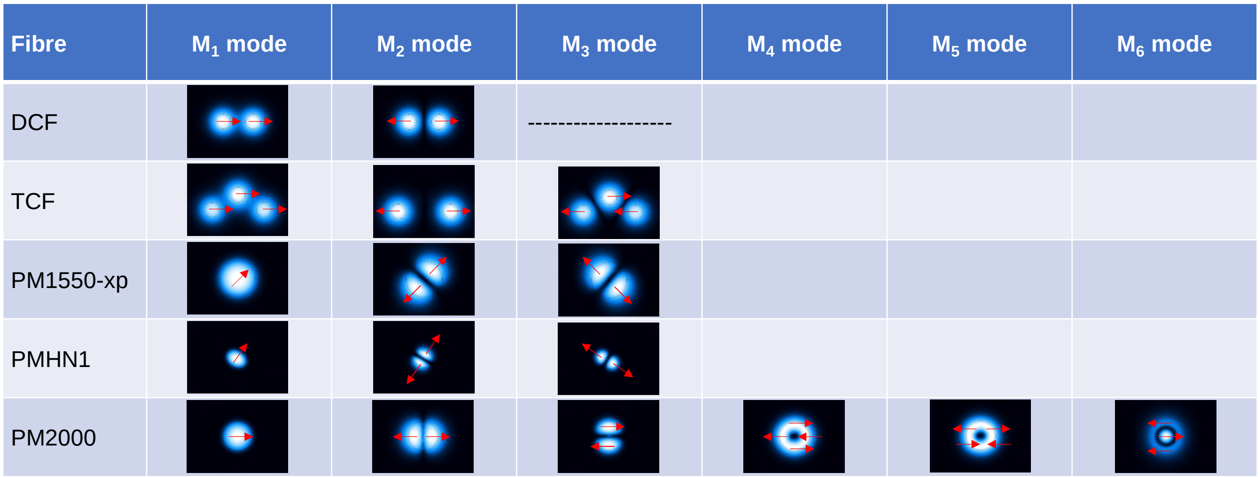

Fig. S1. Modes of the fibres under test. Spatial distribution (intensity) computed from finite element method simulations.

Table S1: Kerr coefficients

Fibre	γ_{11}(W^{-1}km^{-1})	γ_{22}(W^{-1}km^{-1})	γ_{33}(W^{-1}km^{-1})	$\gamma_{12}=\gamma_{21}$(W^{-1}km^{-1})	$\gamma_{13}=\gamma_{31}$(W^{-1}km^{-1})	$\gamma_{23}=\gamma_{32}$(W^{-1}km^{-1})
DCF	3.00	3.12	---	3.06	---	---
TCF	2.30	3.17	2.46	1.56	2.37	1.61
PM1550-xp	2.71	2.12	2.20	1.53	1.52	0.71
PMHN1	13.20	12.50	10.71	8.41	7.32	3.84
PM2000	γ_{11}=5.30 W^{-1}km^{-1}	γ_{22}=4.99 W^{-1}km^{-1}	γ_{33}=4.99 W^{-1}km^{-1}	γ_{44}=2.71 W^{-1}km^{-1}	γ_{55}=2.71 W^{-1}km^{-1}	γ_{66}=2.73 W^{-1}km^{-1}

	γ_{12}=3.41 $W^{-1}km^{-1}$	γ_{13}=3.41 $W^{-1}km^{-1}$	γ_{14}=2.14 $W^{-1}km^{-1}$	γ_{15}=2.12 $W^{-1}km^{-1}$	γ_{16}=2.76 $W^{-1}km^{-1}$	γ_{23}=2.11 $W^{-1}km^{-1}$
	γ_{24}=2.88 $W^{-1}km^{-1}$	γ_{25}=2.87 $W^{-1}km^{-1}$	γ_{26}=1.34 $W^{-1}km^{-1}$	γ_{34}=2.81 $W^{-1}km^{-1}$	γ_{35}=2.80 $W^{-1}km^{-1}$	γ_{36}=1.41 $W^{-1}km^{-1}$
	γ_{45}=2.71 $W^{-1}km^{-1}$	γ_{46}=1.23 $W^{-1}km^{-1}$	γ_{56}=1.22 $W^{-1}km^{-1}$			

Table S2: Inverse group velocity v_n^{-1} of mode-n

Fibre	v_1^{-1} (ns/m)	$v_2^{-1}-v_1^{-1}$ (ps/m)	$v_3^{-1}-v_1^{-1}$ (ps/m)	$v_4^{-1}-v_1^{-1}$ (ps/m)	$v_5^{-1}-v_1^{-1}$ (ps/m)	$v_6^{-1}-v_1^{-1}$ (ps/m)
DCF	4.906	1.068	------	------	------	------
TCF	4.906	0.714	1.513	------	------	------
PM1550-xp	4.895	0.452	0.516	------	------	------
PMHN1	4.978	11.919	0.744	------	------	------
PM2000	4.930	6.517	6.567	8.349	8.467	-6.932

Supplementary Information 2: Mode manipulation via probe-BCB relative polarization

The relative polarization between input probe and BCB represents a further parameter to all-optically reconfigure the output probe. Fig. S2 illustrates some experimental results in the case of a bimodal fibre (DCF). The evolution of the output probe mode distribution as a function of the BCB power is reported when input probe and BCB are either co-polarized (Fig. S2a) or orthogonally polarized (Fig. S2b) and while maintaining the same input probe and BCB mode state. In the case of orthogonal polarization, we observe a slower modal conversion dynamic, due to the weaker interaction between the probe and the BCB (coefficient κ is reduced by a factor of 1/3 in equation (6) of the manuscript). Fig. S2c shows the mode distribution of the output probe when the probe-BCB relative polarization is continually adjusted from 0 deg (co-polarized) to 90 deg (orthogonally polarized), whereas the BCB power is fixed (8 kW peak-power). We observe that the fraction of the output probe coupled to mode M_1 (M_2) is tuneable in the range 37-75% (30-70%).

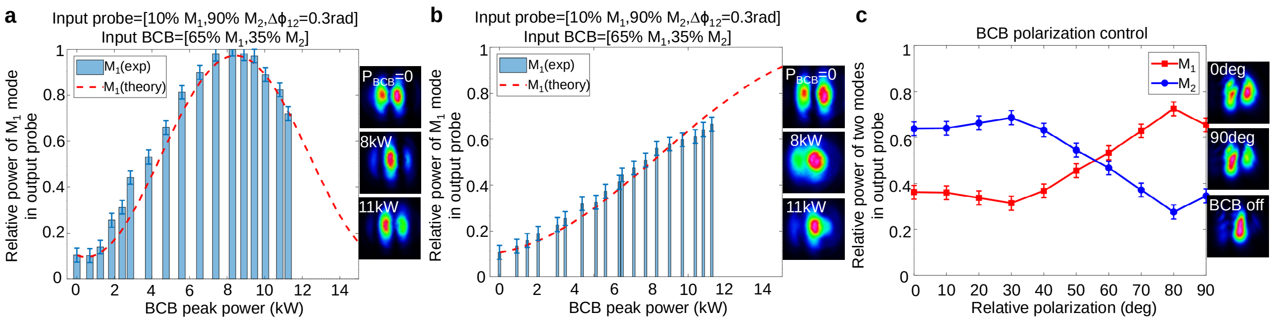

Fig. S2. Tuneable mode manipulation by adjusting the polarization. a,**b**. Comparison between output probe mode distribution in the co-polarized case (panel a) and orthogonally-polarized case (panel b). The insets show the far-field intensity of the output probe at different BCB power P_{BCB}. **c**. Mode distribution of the output probe as a function of the probe-BCB relative polarization for a fixed BCB power (8kW peak power). The insets show the far-field intensity at 0 deg, 90 deg and when BCB is turned off.

Supplementary Information 3: Ultrafast dynamics

In our experiments, we have demonstrated that the core-to-core power ratio of the output probe can be switched on a sub-nanosecond timescale (see Fig. 5d,e of the manuscript).

In the following, we discuss some numerical results that illustrate the core-to-core switching mechanism in detail. These results are obtained by simulation of the full CNLSEs reported in equation (6) of the manuscript. We focus on the case of pulsed BCB. For simplicity, we illustrate the case of a dual core fibre with L=1 m, $\gamma_{12}=1/\mathrm{W/km}, \gamma_{11}=\gamma_{22}=2/\mathrm{W/km}$. The system under analysis possesses three critical timescales: the BCB pulse width τ_P; the BCB repetition rate R; and the time of flight $\tau_F=L/c$ in the fibre (c= light velocity in the fibre). In

these simulations, the BCB is equally distributed in the 2 fibre modes and the BCB pulse width τ_p=1 ps. Full core-to-core conversion (from 100/0 to 0/100) in the output probe is achieved for a BCB peak power P_{BCB} =6.2 MW. The probe peak power is instead arbitrary low.

Initially, we consider the case of a continuous-wave probe. In Fig. S3, we show the output probe temporal dynamics when the BCB is respectively off (Fig. S3b) and on (Fig. S3c). When the BCB is off, the output probe power is fully coupled to core 1 (100/0 power ratio). When the BCB is on, the first BCB pulse with P_{BCB} =6.2 MW (BCB pulse 1) triggers the full switching of the output probe power ratio from 100/0 to 0/100. This state is maintained over a time window $2\tau_F = 2L/c$, after which the power ratio returns to 100/0 (BCB off condition).

At the following BCB pulse (BCB pulse 2), after a time 1/R, the dynamics repeat. However, the peak power of BCB pulse 2 is now lower, i.e. 3.2 MW, resulting in a reduced conversion (45/55 power ratio). A similar dynamic applies to BCB pulse 3, whose low power results in a weak conversion (80/20 power ratio). Note that if the condition 1/R =$2\tau_F$ is met, then it is possible to maintain the output probe power ratio 0/100, as shown in Fig. S4. Similar considerations apply when the probe is pulsed: regardless of the probe pulse width, each probe pulse within a time window of width $2\tau_F$ is switched, as reported in Fig. S5.

It is worth noting that the results illustrated above are generalizable to fibres with different parameters and/or more cores, as well as different pulse widths. Similarly, these results would extend to other waveguide systems beyond optical fibres. For example, using silicon-based integrated waveguides (which possess nonlinearities > 3 orders of magnitude higher than standard optical fibres), similar results to Figs. S3-S5 could be achieved but reducing the peak powers in the fraction-of-kW range. These peak power levels, along with ps pulses and GHz repetition rates, can be currently obtained using commercial fibre lasers. The combination of innovative all-optical pulse generation techniques (see Ref. S1) with our all-optical switching approach would underpin the development of an ultrafast all-optical integrated switching system.

Note finally that the switching time τ_{switch}, required to complete the core-to-core switching, is of the order of the BCB pulse width τ_p (see insets of Fig. S3). The latter is ultimately constrained by distortion due to interplay among dispersion and self-phase modulation, which poses a limit to the minimum BCB pulse width.

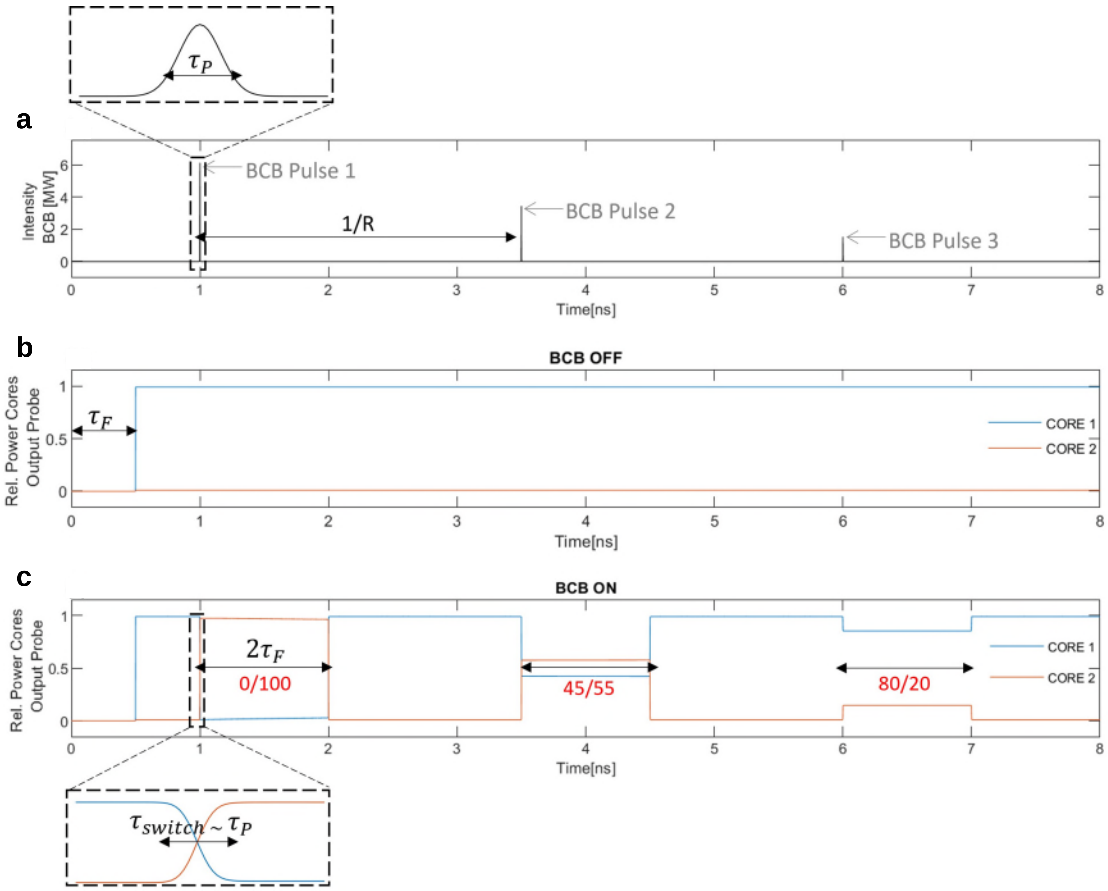

Fig. S3. Ultrafast dynamics with pulsed BCB, CW probe. Each BCB pulse (see panel a and related inset) causes a switching in the output probe core-to-core power ratio. The achieved power ratio (here 0/100, 45/55 and 80/20 for BCB pulses 1, 2 and 3, respectively) depends on the BCB pulse peak power. The switching is preserved over a time window of length $2\tau_F$, where the time of flight τ_F is shown in panel b.

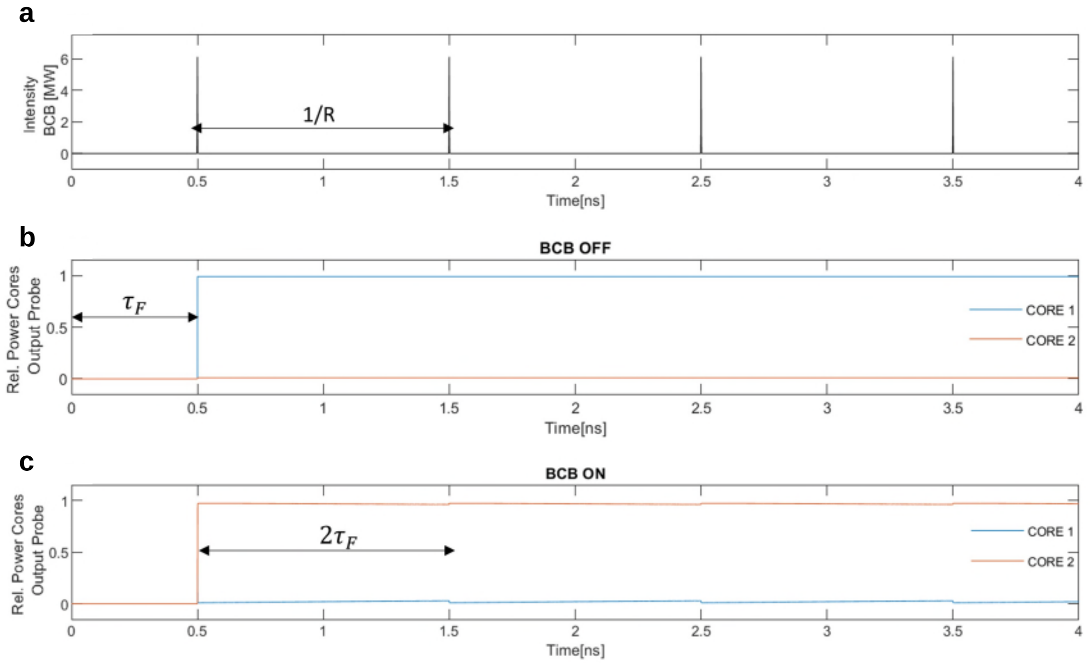

Fig. S4. Preserving 0/100 state. As Fig. S3, but here each BCB pulse has peak power 6.2 MW and the condition 1/R $=2\tau_F$ is met. Consequently, the output probe core-to- core power ratio 0/100 is preserved.

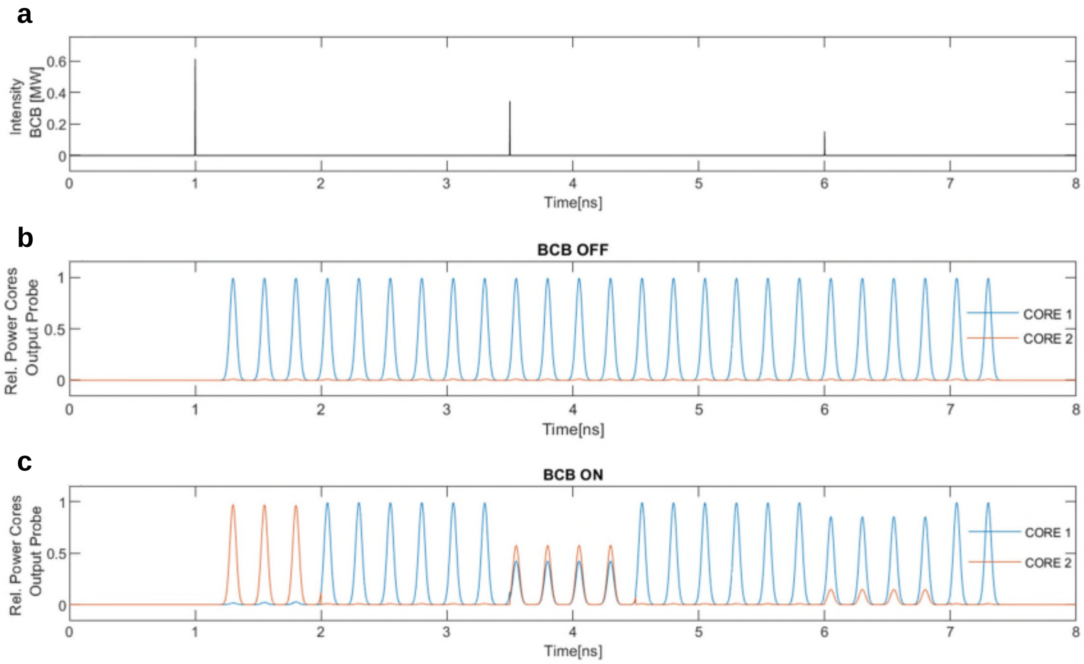

Fig. S5. Ultrafast dynamics with pulsed BCB, pulsed probe. As Fig. S3, but here the probe is pulsed with pulse width =200ps.

Supplementary Information 4: temporal and spectral characterization of the BCB

We present in Fig. S6 experimental measurements of the backward control beam (BCB) in both the time and frequency domains, recorded at the fibre input and output for the highest peak power level (11 kW) used in our experiments with the DCF. These measurements confirm that no significant detrimental effects—such as Raman and Brillouin scattering, four-wave mixing-induced wavelength conversion, self-phase modulation (SPM), or cross-phase modulation (XPM)-induced pulse reshaping and spectral broadening—are observed. The spectral shape of the BCB remains nearly unchanged between input and output, indicating negligible nonlinear spectral broadening and wavelength conversion. Similarly, the temporal profile exhibits no noticeable distortion, confirming that the BCB pulse propagates through the fibre without significant reshaping. Similar results are obtained for all the fibres under test.

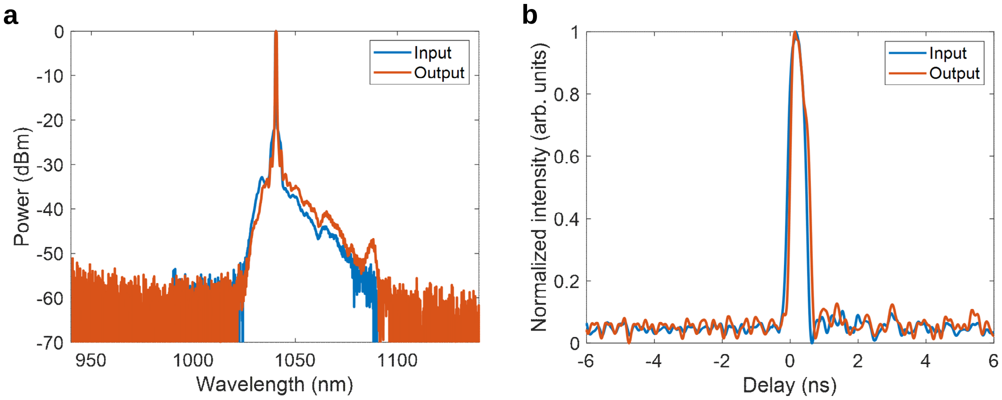

Fig. S6. Temporal and spectral measurements of the BCB in the DCF for the highest peak power level used. a. Input and output BCB spectrum. **b.** Input and output temporal pulse.

Supplementary Information 5: robustness of our remote sensing approach

As reported in the manuscript (section Results, Application 3), in the absence of mode coupling, the standard transfer matrix approach would estimate the input relative phase $\Delta\phi_{in,12}$ between two modes as:

$$\Delta\phi_{in,12} = \Delta\phi_{out,12} - \Delta\phi_{acc} \quad \text{(S1)}$$

Even under the ideal assumption of a perfect measurement of the output phase $\Delta\phi_{out,12}$, this approach is fundamentally limited by the accumulated phase delay $\Delta\phi_{acc} = \Delta\beta_{12}L$, where L is the fibre length and $\Delta\beta_{12}$ is the differential propagation constant between the modes, which is highly sensitive to external perturbations.

The error in the transfer matrix approach arises from the fact that the pre-computed phase accumulation term, $\Delta\phi_{acc}$, is typically obtained under reference conditions—such as a reference temperature T_{ref} (e.g., 0°C) or a reference bending radius $r_{B,ref}$ (e.g., infinity, meaning a straight fibre). However, in practical scenarios, the actual temperature T and bending radius r_B inevitably deviate from these reference values.

Consider, for example, a temperature variation. The actual relative input phase at temperature T is given by:

$$\Delta\phi_{in,12}(T) = \Delta\phi_{out,12}(T) - \Delta\phi_{acc}(T) \quad \text{(S2)}$$

However, using the transfer matrix approach, the estimated relative input phase reads:

$$\Delta\widetilde{\phi}_{in,12}(T) = \Delta\phi_{out,12}(T) - \Delta\phi_{acc}(T_{ref}) \quad \text{(S3)}$$

This leads to an error per unit length:

$$(\Delta\phi_{in,12}(T) - \Delta\widetilde{\phi}_{in,12}(T))/L = \Delta\beta_{12}(T) - \Delta\beta_{12}(T_{ref}) \quad \text{(S4)}$$

Similarly, in our approach, the error arises when estimating $\Delta\phi_{in,12}(T)$ by Eq. (4) while using Kerr coefficients precomputed at T_{ref}. A similar reasoning applies to errors induced by fibre bending variations.

However, $\Delta\beta_{12}$ is strongly sensitive to perturbations, for example, Fig. S7a,b presents $\Delta\beta_{12}$ as a function of temperature and bending radius for one of the fibres under test, namely PM1550-xp (mode 1 is LP_{01}; mode 2 is LP_{11e}). In contrast, the Kerr coefficients remain nearly unaffected by these perturbations, see Fig. S7c,d.

This distinction has a profound impact on the overall estimation error. Since variations in $\Delta\beta_{12}$ directly affect $\Delta\phi_{in,12}$ when using the transfer matrix approach, the transfer matrix approach becomes highly unreliable. As shown in Fig. S8a, a temperature variation of just a few degrees over a few meters of fibre can induce a phase error of π rad, rendering the phase estimation from the transfer matrix approach entirely inaccurate. Conversely, the near-invariance of the Kerr coefficients ensures a substantial suppression of estimation errors in our approach (<0.001 rad/m), as confirmed by the data in Fig. S8c,d.

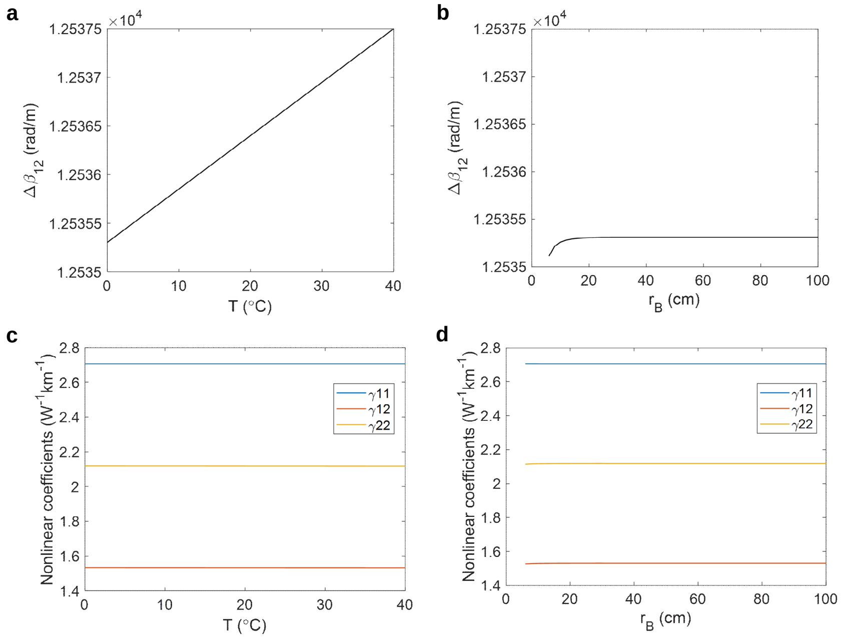

Fig. S7. Fibre parameters versus temperature and bending. Finite element simulations. a,b. Differential propagation constant $\Delta\beta_{12}$ between modes LP_{01} and LP_{11e} in the PM1550-xp fibre, versus temperature T and fibre bending radius r_B. To account for temperature

variations, a uniform refractive index variation dn = 1×10^{-5}/K across both the core and cladding of the fibre is assumed (Ref. S2). **c,d.** Same as a,b but for the Kerr coefficients (γ_{11}, γ_{12}, and γ_{22}).

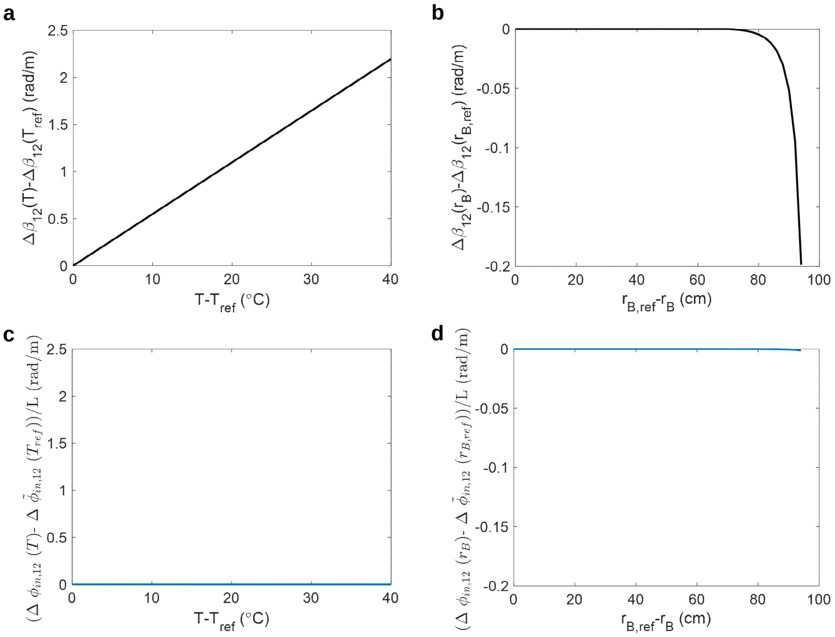

Fig. S8. Estimation errors: transfer matrix vs our approach. a,c. Error per unit length $(\Delta\phi_{in,12}(T) - \Delta\widetilde{\phi}_{in,12}(T))/L$ when T_{ref} =0 °C in the transfer matrix method (a) and our approach (c). **b,d.** Error per unit length $(\Delta\phi_{in,12}(r) - \Delta\widetilde{\phi}_{in,12}(r))/L$ when bend radius $r_{B,ref}$ = 100 cm in the transfer matrix method (b) and our approach (d).

References

S1. Berti, N., Coen, S., Erkintalo, M. & Fatome, J. Extreme waveform compression with a nonlinear temporal focusing mirror. *Nat. Photonics* **16**, 822-827 (2022).

S2. N. Shibata, S. Shibata, and T. Edahiro. Refractive index dispersion of lightguide glasses at high temperature. *Electronics letters* **17**, 310-311 (1981).
