# Supplementary material for: Sub-nanosecond all-optically reconfigurable photonics in optical fibres
Source: Nat Commun. 2025 Jul 19;16:6665. doi: 10.1038/s41467-025-61984-8 (PMC12276229; doi:10.1038/s41467-025-61984-8)
Supplement: Supplementary file 2 — Description of Additional Supplementary Files [file 41467_2025_61984_MOESM2_ESM.pdf]

Description of Additional Supplementary Files

Supplementary Movie 1:

Measured and reconstructed far-field of the output probe as a function of the BCB peak power. Fibre parameters and mode decomposition are those related to Fig. 3d in the manuscript.

Supplementary Movie 2:

Measured and reconstructed far-field of the output probe as a function of the BCB peak power. Fibre parameters and mode decomposition are those related to Fig. 3e in the manuscript.

Supplementary Movie 3:

Measured and reconstructed far-field of the output probe as a function of the BCB peak power. Fibre parameters and mode decomposition are those related to Fig. 3f in the manuscript.
